# CSGene: a literature-based database for cell senescence genes and its application to identify critical cell aging pathways and associated diseases

**DOI:** 10.1038/cddis.2015.414

**Published:** 2016-01-14

**Authors:** M Zhao, L Chen, H Qu

**Affiliations:** 1 School of Engineering, Faculty of Science, Health, Education and Engineering, University of the Sunshine Coast, Maroochydore DC, Queensland 4558, Australia; 2 Center for Bioinformatics, State Key Laboratory of Protein and Plant Gene Research, College of Life Sciences, Peking University, Beijing 100871, People's Republic of China

## Abstract

Cell senescence is a cellular process in which normal diploid cells cease to replicate and is a major driving force for human cancers and aging-associated diseases. Recent studies on cell senescence have identified many new genetic components and pathways that control cell aging. However, there is no comprehensive resource for cell senescence that integrates various genetic studies and relationships with cell senescence, and the risk associated with complex diseases such as cancer is still unexplored. We have developed the first literature-based gene resource for exploring cell senescence genes, CSGene. We complied 504 experimentally verified genes from public data resources and published literature. Pathway analyses highlighted the prominent roles of cell senescence genes in the control of rRNA gene transcription and unusual rDNA repeat that constitute a center for the stability of the whole genome. We also found a strong association of cell senescence with HIV-1 infection and viral carcinogenesis that are mainly related to promoter/enhancer binding and chromatin modification processes. Moreover, pan-cancer mutation and network analysis also identified common cell aging mechanisms in cancers and uncovered a highly modular network structure. These results highlight the utility of CSGene for elucidating the complex cellular events of cell senescence.

Cell senescence refers to the state of permanent cell-cycle arrest when cells respond to exogenous and endogenous stress signals.^1^ Different from other nondividing cellular events, cell senescence is characterized by a lack of proliferative activity and DNA damage markers. Cell senescence often has nuclear foci of constitutive heterochromatin.^2^ The gene expression of tumor suppressors and cell-cycle inhibitors is also absent.^2^ In addition, senescent cells secrete signaling molecules to change the tissue microenvironment.^2^

The importance of cell senescence was first recognized in normal human embryonic fibroblasts by Hayflick and Moorhead in 1961.^3^ They found that fibroblast cells lost proliferative capacity after numerous *in vitro* cultures. This limited replication phenomenon overturned the theory of cellular immortalization. Moreover, Hayflick proposed the existence of a finite division limit for cells. Cell senescence has both beneficial and detrimental roles in cell development.^2^ On one hand, cell senescence contributes to eliminating damaged cells, and on the other hand, persistent senescence may cause loss of cell-proliferative and tissue-regenerative capacities and eventually lead to cell death.

Recently, the relationship between cell senescence and cancer has become increasingly clear from numerous studies.^4^ Cell senescence is considered to be prevalent in premalignant cancer cells and a critical barrier against cancer progression to malignancy.^5^ Generally, the senescent cancer cells can be recognized and cleaned efficiently by immune cells.^4^ However, recent evidence also indicates that cell senescence can also promote cancer cell proliferation.^6^ In additionally, it has recently been found that the senescence signal can also initiate microRNA-directed gene silence that may make the cell senescence process more complex.^7, 8^ Therefore, questions still remain regarding (1) what the complex molecular network is for a tumor cell to evade cell senescence; (2) how cell senescent-related genes have dual roles in cancer progression as tumor suppressors and oncogenes; and (3) what the translational significance of cell senescence is to clinical oncology.

Although cell senescence is essential in our understanding of tumor suppression mechanisms, genetic and biological information of cell senescence is sparse in the literature. To the best of our knowledge, there has been no systematic review published to provide understanding of the molecular blueprint of cell senescence in multiple cancer types. A high-quality gene database is needed to further broaden the molecular picture for cell senescence and associated diseases. In this study, we developed the first literature-based cell senescence gene database, CSGene, that serves as a reference data set for understanding the cellular mechanisms of cell senescence processes during cancer progression. The resultant gene list in CSGene and additional annotations will be a valuable resource for cell senescence and related biomedical research communities.

## Results

### Data integration and literature search

**Figure 1:**
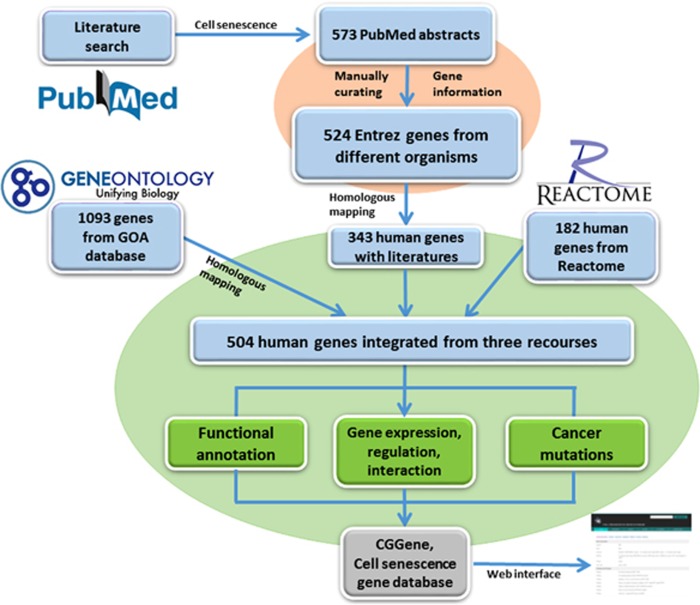
Pipeline for collection and annotation of cell senescence genes

The primary aim of the CSGene database is to collect and maintain a high-quality cell senescence gene resource to serve as a comprehensive, classified, and accurately annotated knowledgebase for cell senescence. To provide a comprehensive resource, we collected known cell senescence-related genes from the Reactome pathway database,^9^ the Gene Ontology database,^10^ and the PubMed/GeneRIF literature database^11^ (Figure 1). To retrieve a comprehensive list of annotated genes from the Gene Ontology annotation database (GOA), we curated four GO terms related to cell senescence: cellular senescence (GO:0090398), stress-induced premature senescence (GO:0090400), oncogene-induced cell senescence (GO:0090402), and oxidative stress-induced premature senescence (GO:0090403). We then extracted 1093 proteins from various organisms in the GOA database associated with cell senescence GO terms on 8 January 2015.

We also downloaded 182 human genes related to cell senescence from the Reactome database. These 182 human genes are annotated on the following pathways: Cellular Senescence (pathway ID: 905991), Cellular Responses to Stress (pathway ID: 645258), Oxidative Stress-Induced Senescence (pathway ID: 905993), Senescence-Associated Secretory Phenotype (SASP) (pathway ID: 905996), DNA Damage/Telomere Stress-Induced Senescence (pathway ID: 905994), and Oncogene-Induced Senescence (pathway ID: 905992). However, neither the GOA nor the Reactome databases provide original literature to support the roles of cell senescence genes, and the data curation is slower than the pace of cell senescence biology research.

To provide a detailed and precise cell senescence gene resource with literature evidence, we performed an extensive literature query on the GeneRIF database on 10 January 2015, using the keywords ‘cell' and ‘senescence' that returned 878 GeneRIF records associated with 573 PubMed abstracts. GeneRIF (Gene Reference Into Function) is a collection of records with short descriptions about gene function in the Entrez Gene database.^11^ To further curate the matching literature, we downloaded all 573 PubMed abstracts in a Medline format for manual review.

Curation of cell senescence genes from the literature included three major steps. (1) The 573 abstracts retrieved were highlighted with the keywords ‘cell' and ‘senescence' and grouped by a function in the Entrez system in related articles. This step arranges the abstracts related to similar genes together. (2) An in-house Perl script was implemented to extract the sentences with the keywords ‘cell' and ‘senescence' from grouped abstracts. As the grouped abstracts are closely related, this step allowed us to quickly and easily cross-check and evaluate whether a curated abstract was related to cell senescence genes and how they were related. (3) We further manually collected gene names from the text of the cell senescence genes.

To gain precise cell senescence information, much care was taken regarding species information and gene name/alias. For example, consider the following sentence: ‘*The SIRT1-PARP-1 axis plays a critical role in the regulation of cigarette smoke(CS)-induced autophagy and has important implications in understanding the mechanisms of CS-induced cell death and senescence*'.^12^ Both genes *Sirt1* and *Parp1* were collected and mapped to Gene IDs of ‘mouse' in the current Entrez gene database. We used Entrez gene IDs for cell senescence genes to serve as the database key to crosslink the same genes from different public databases. After carefully checking them manually, we pinpointed 524 Entrez genes from various species from 564 PubMed abstracts. To provide a consistent overview, we mapped all 524 genes to 342 human genes using homologous relationships from the NCBI HomoloGene database, as we have done previously, that provided cross-checking among multiple species.^13, 14, 15^ By integrating the cell senescence genes from GOA and Reactome, we consolidated the 504 human genes shown in Supplementary Table S1 (480 protein-coding and 23 noncoding genes). This literature-based gene list will be updated regularly based on newly published literature. Using the resulting human genes, we retrieved 5115 homologs from other species in the HomoGene database.

### Pathway enrichment analysis revealed the fundamental role of cell senescence in rRNA gene transcription

**Figure 2:**
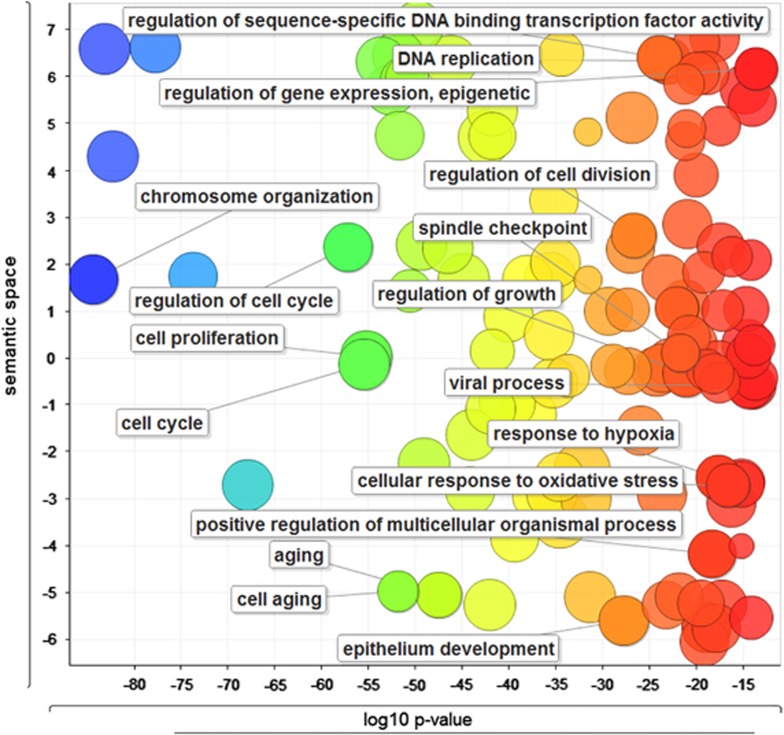
Enriched Gene Ontology terms for 504 human cell senescence genes. The scatterplot shows the GO clusters for the 504 human cell senescence genes in a two-dimensional space derived by applying multidimensional scaling to a matrix of the GO terms' semantic similarities. The x axis represents the logarithm of correlated *P*-value, and the y axis represents the similarity between the enriched Gene Ontology terms. Bubble color represents the logarithm of corrected *P*-value (the smaller corrected *P*-value is toward red color). Bubble size indicates the frequency of the GO term in the GOA database (the more general the term, the larger the bubble)

Functional enrichment analyses have been widely used to identify classes of genes or proteins that are overrepresented in any interesting biological functions or phenotype.^16^ We used the implemented pathway enrichment analysis to characterize whether the cell senescence genes had a different frequency of annotation pairs that was unlikely to have occurred by chance, given all the human genes as the background. As shown in Figure 2, a quick Gene Ontology enrichment analysis revealed that those human cell senescence genes are involved in many housekeeping functions in a cell. These functions are mainly associated with cell division, cell cycle, and cell proliferation.

As shown in Table 1, the enriched biological pathways are also expected to be related to cell-cycle, meiosis, and telomere functions (the full pathway list is shown in Supplementary Table S2). However, the top-ranked pathway is about ‘RNA Polymerase I Promoter Opening' (corrected *P*-value=3.79E−79). In addition, there are three other pathways about RNA polymerase I transcription (corrected *P*-value=1.15E−62), elongation (corrected *P*-value=1.59E−62), and promoter clearance (corrected *P*-value=5.88E−62). RNA polymerase I mainly synthesizes ribosomal RNA (but not 5S rRNA that is transcribed by RNA polymerase III), a type of RNA that accounts for >50% of the total RNA synthesized in higher eukaryotic cells.^17^ This is the first report to associate RNA polymerase I activity with cell senescence. However, recent studies indicate that the ribosomal RNA genes contain highly repetitive structures.^18^ These repeats are the most fragile sites in the chromosome.^18^ Moreover, the unstable features of rDNA in the genome can accelerate cellular senescence.^19^ As a center of genome integrity maintenance, the repeat number of rDNA can determine sensitivity to DNA damage. Combining all these results, CSGene may provide a useful resource for exploring the cell aging processes related to rDNA synthesis.

The other interesting pathways include ‘Amyloids', ‘Alcoholism', and ‘Progesterone-mediated oocyte maturation'. Amyloids are insoluble fibrous protein fragments that the body produces normally. However, amyloids may inappropriately fold and erroneously interact with other cell components to form insoluble fibrils. The aggregates of fibrils may lead to amyloidosis and numerous human neurodegenerative disorders.^20^ As a neurotoxin, alcohol abuse may trigger aging processes, hypertension, cardiac dysrhythmia, cancers, gastrointestinal disorders, neurocognitive deficits, bone loss, and emotional disturbances, especially depression.^21^ However, it is still not clear how alcohol metabolism connects with cell senescence at a molecular level. CSGene may provide a link to explore the potential cellular mechanism of alcoholism in cell aging. In summary, cell senescence may have fundamental roles in genome stabilities, metabolism of amyloids, and alcoholism that may deserve further exploration on systematic level such as network analysis.^22, 23, 24^

### Chromatin modification-related cell senescence genes are overrepresented in HIV-1 infection, viral carcinogenesis, and multiple cancer types

With a fundamental role in cell destination, it is not surprising that the cell senescence genes are consistently associated with a number of human diseases. Gene-set-based enrichment analysis may allow for testing of the risk factor of cell senescence in complex diseases. As shown in Table 2, the 504 cell senescence genes are related to a broad spectrum of human diseases, such as cancers (Supplementary Table S3 has the full list), that is expected. In total, there are 272 cell senescence genes associated with various cancer types (Supplementary Table S4). The cancers mainly occur in the circulatory, digestive, and urinary systems, including the bladder, blood, colon, endometrium, kidney, melanoma, ovary, pancreas, prostate, stomach, and rectum (all corrected *P*-values <0.01). We also found 68 genes involved in viral carcinogenesis (corrected *P*-value=9.942E−40).

It is also interesting to note that the 504 cell senescence genes are overrepresented in processes related to HIV-1, systemic lupus erythematosus, and viral carcinogenesis (Table 2 and Supplementary Table S4). The possible association of cell senescence with HIV infection is also supported by acceleration of the adaptive immune system aging process.^25^ It is known that the immunological features of HIV-1-infected patients have similar symptoms to elderly people without HIV infection. However, the detailed molecular mechanisms are not clear. We found 56 genes in the CSGene database associated with numerous chromatin regulatory processes in HIV infection (Supplementary Table S4), including ‘Transcriptional activation of the integrated chromatin-associated human immunodeficiency virus type 1 promoter', ‘Regulation of HIV-1 gene expression by histone acetylation and factor recruitment at the LTR promoter', ‘Enhancement of the p300 HAT activity by HIV-1 Tat on chromatin DNA', and ‘Acetylation of HIV-1 Tat by CBP/P300 increases transcription of integrated HIV-1 genome and enhances binding to core histones' (Table 2). Combining these observations, one of the driving molecular mechanisms for cell aging may be active chromatin modifications.^26^

**Figure 3:**
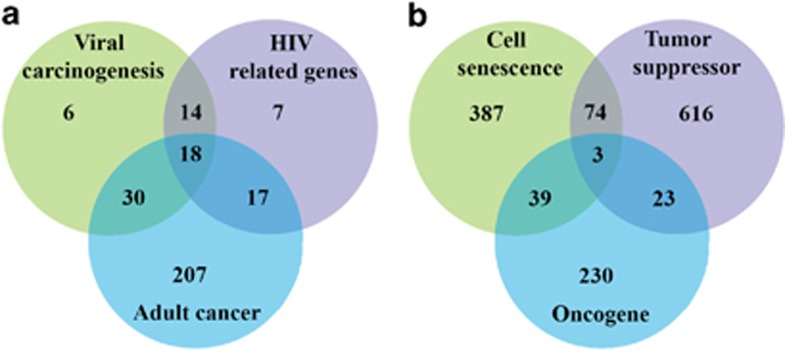
The overlapping of cell senescence genes with HIV infection-related genes, viral carcinogenesis-related genes, cancer-related genes, tumor suppressors, and oncogenes. (**a**) Venn diagram for distribution of cell senescence genes involved in HIV infection, viral carcinogenesis, and various adult cancers. (**b**) Venn diagram for human cell senescence genes, tumor suppressors, and oncogenes

To further explore the common mechanisms for cell senescence in HIV infection, viral carcinogenesis, and cancer, a Venn diagram was generated to identify the shared cell senescence genes (Figure 3a). Surprisingly, 17 of the 18 common genes from three types of diseases are histone proteins. In addition, there are 13 coding an H4 component. Previous evidence showed that the trimethylation of histone H4 at lysine 20 (H4K20me3) increases in rat liver with age and is upregulated in a cellular model of progeria.^27, 28^ These overwhelming H4 and histone proteins are shared by three cell senescence-related diseases and confirm that H4 is related to aging, and H4 histone modifications are a hallmark of constitutive heterochromatins linked to aging processes.

### Ranking the important genes in cell senescence and their high frequency of mutations in multiple SCCs

As one of the fundamental cellular process, hundreds of genes associated with cell senescence reflect the complexity of genetic organization. Small-scale studies about cell senescence often focus on verifying specific functions under a certain stressful cellular environment. Therefore, there is no systematic selection of the most informative genes or big map constructed for cell senescence. Using the gene ranking tool ToppGene, we obtained the top 10 ranked senescence-related genes: *CTNNB1*, *SMAD3*, *HIF1A*, *TGFB1*, *ZEB2*, *EGFR*, *CDH1*, *ILK*, *ZEB1*, and *TWIST1* (Table 3; Supplementary Table S5 has the full list). Not surprisingly, a majority of these top-ranked genes are involved in the key pathways of cell senescence, such as the ‘Pathways in cancer', ‘Adherens junction', ‘Focal adhesion', and ‘TGF-beta signaling pathway'.

Although cellular senescence is an important mechanism for suppressing tumorigenesis, the somatic mutational pattern across multiple cancer types has not been systematically examined. It is useful for users to identify potential tumor suppressor genes for further screening. By overlapping the known tumor suppressor genes with oncogenes, we found that cell senescence-related genes may have roles in tumor suppression or cancer progression (Figure 3b). Thus, a precise cancer suppressing strategy from a cell senescence angle may consider the dual roles of cell senescence genes.

**Figure 4:**
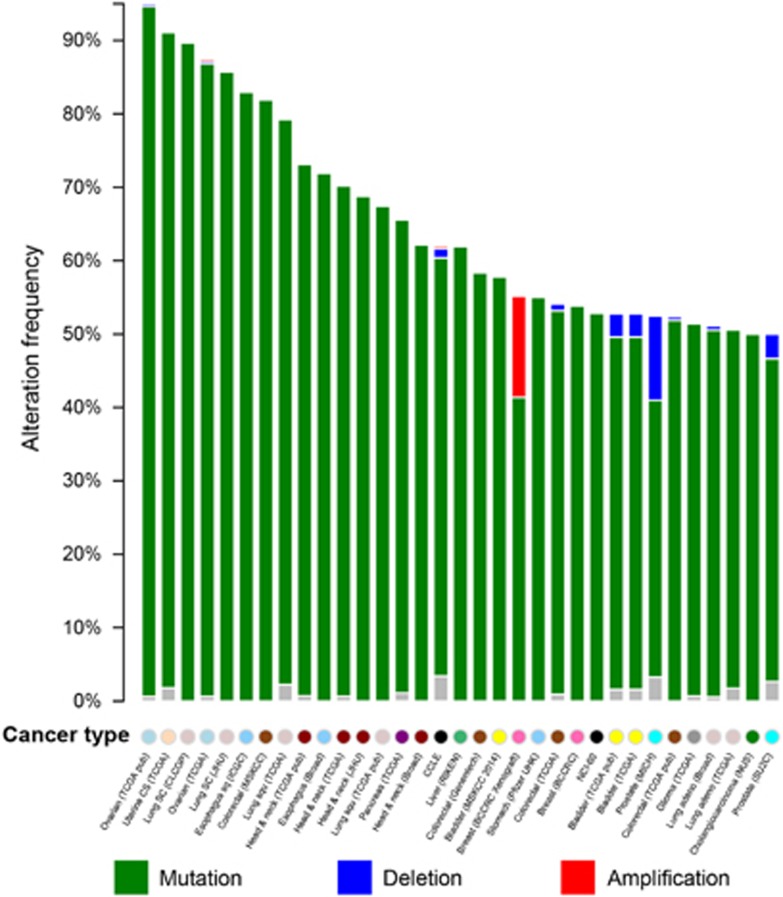
Mutational landscape for the top 100 ranked cell senescence genes in multiple cancers. The green, blue, and red bars represent the percentage of the single-nucleotide mutation, gene copy deletion, and amplification in cancer samples, respectively. The gray bar indicates the percentage of multiple genetic mutations (single-nucleotide mutation, gene copy deletion, or amplification) in the cancer samples

To further explore the mutational pattern for the most important cell senescence genes, the top 100 ranked cell senescence genes were mapped to public cancer genomics data (Figure 4). Interestingly, we found that these genes have mutated in over 50% cases of 33 cancer data sets (Supplementary Table S6). In addition, all these mutations are almost single-nucleotide mutations with no deletion or amplification. For example, the 100 genes have single-nucleotide mutations among 94.90% of 300 individuals with ovarian serous adenocarcinoma. A similar prevalence of single-nucleotide mutations can be found in all top 13 mutated cancer studies for 7 cancer types: colorectal adenocarcinoma, esophageal squamous cell carcinoma (SCC), lung SCC, small-cell lung cancer, head and neck SCC, uterine carcinosarcoma, and ovarian serous adenocarcinoma. Interestingly, seven of these cancer studies are on SCC. These seven studies all have mutational rates ranging from 62.2 to 83%. In contrast, the remaining 76 studies have mutation rates of ^<^62.2% of the samples (Supplementary Table S6), and none of them were purely on SCC. This suggests that SCC may have distinct mechanisms related to cell senescence, in contrast to other carcinomas. For cancers with abundant mutations in cell senescence genes, cellular senescence may provide a feasible tumor suppression mechanism *in vivo* that may be new avenue for developing novel therapeutics for these devastating cancers.

**Figure 5:**
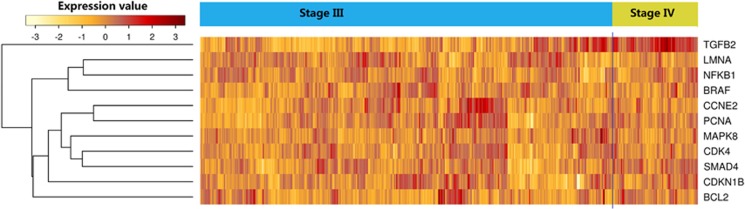
The heatmap for the 11 genes differentially expressed between stages III to stage IV in TCGA ovarian cancer samples

As the top 100 genes are highly mutated in 300 samples from TCGA (The Cancer Genome Atlas) ovarian serous cystadenocarcinoma,^29^ we further explored the differentially expressed genes for those samples in different cancer stages. The TCGA ovarian serous cystadenocarcinoma study generated a gene expression profile of 489 samples. However, 460 of these samples are from stages III and IV (94%). As shown in Figure 5, we identified that 11 cell senescence-related genes are differentially expressed along the transition from stages III and IC. Among these genes, *CDKN1B* can be both tumor suppressor and oncogene according to TSGene (tumor suppressor gene) database.^14^ The *BLC2* and *BRAF* are oncogenes whereas *SMAD4* and *NFKB1* are tumor suppressors.^14^ This analysis may provide an overview for cell senescence-related genes in different cancer stages with different senescent features.

### The reconstructed cell senescence PPI network exhibits a highly modular structure

**Figure 6:**
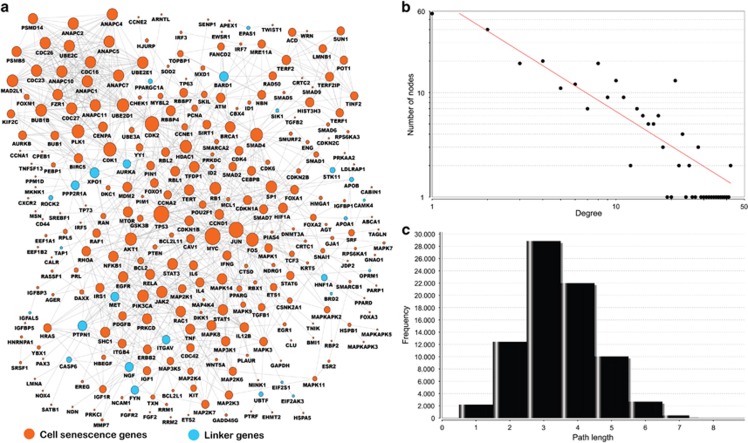
Reconstructed cell senescence map using protein–protein interaction data. (**a**) The 260 genes in orange are from the core data set in CSGene. The remaining 26 genes in blue are linker genes that bridge the 260 genes. (**b**) Degree distribution in the reconstructed map. (**c**) The distribution of short path length in the reconstructed map

Recent advances in high-throughput technologies have dramatically increased the availability of protein–protein interaction (PPI) data and have stimulated pathway reconstruction for improving the systems-level understanding of specific cellular events. To avoid a high level of noise, sparseness, and highly skewed degree distribution of physical interaction-based PPI networks, we used only reliable human PPIs summarized in a few popular biological pathway databases, such as KEGG and the Reactome pathway database.^30^ Using a module searching method,^31^ we extracted a subnetwork from all human pathway-based interactomes. The reconstructed cell senescence network contains 286 genes and 1099 gene–gene interactions based on current evidence from known biological pathways (Figure 6a). Of the 286 nodes, 260 are from our 504 curated cell senescence genes. The remaining 26 nodes are novel genes that may potentially allow the cell senescence genes to fully implement their cellular function. A majority of cell senescence genes are linked to each other in a highly modular structure. This also supports the accuracy of our data.

Further network topological analysis also indicates that most molecules in our map are closely connected. There are only 57 nodes with one connection (Figure 6b). This means that a majority of nodes have multiple connections. The degrees of all nodes in our reconstructed cell senescence map follow a power law distribution, *P(k)~k*^*−b*^, where *P(k)* is the probability that a molecule has connections with *k* other molecules, and *b* is an exponent with an estimated value of 1.101. This means that our cell senescence map is different from the human PPI network, where most nodes are sparsely connected with a *b-*value of 2.9.^32^ This feature reduces the distribution of shortest path length for the entire network to a smaller number range (2 to 4). It also means that ∼53% of the node communication can be reached in only three steps (Figure 6c). With high modularity, the hub nodes in this network may have prominent roles as common connections to mediate information transduction within a short path. In total, there are 5 genes with at least 30 connections: *TP53* (42), *MYC* (42), *JUN* (38), *CDK2* (35), and *CDK1* (30) (Supplementary Table S7). All of these genes have been reported to be involved in cell senescence. In summary, our reconstructed map not only discovers multiple paths related to a few known signaling pathways but also provides a broader picture for the highly modular structure of previously unconnected cell senescence signaling pathways.

## Discussion

CSGene was constructed as a free database and analysis server to enable users to rapidly search and retrieve summarized cell senescence genes. Our systematic pathway and disease enrichment analyses reveal that the cell senescence genes have important roles in rRNA gene transcription and chromatin modification that are related to genome stability. We believe that CSGene is the first example of an integrated and comprehensive gene resource to help elucidate the relationship between cell senescence and cancers. This database could have profound implications for the diagnosis, treatment, and prevention of diseases related to cell senescence.

The comprehensive functional enrichment analyses reveal that cell senescence genes are enriched in multiple signal events associated with development and cancers. Important questions should now be directed toward the integration of various signaling pathways with initial cell senescence events. CSGene is freely available at http://csgene.bioinfo-minzhao.org/.

Specific to cancer progression, there is also a lack of systematic exploration of the dual roles of cell senescence as tumor suppressor and oncogene. Leveraging the available tumor suppressor and oncogene list from the TSGene database,^14^ we found there are 77 cell senescence-related genes that are tumor suppressor genes and 42 that are oncogenes. This simple overlapping relationship may indicate that cell senescence has dual roles in cancer progression. For certain genes, the genes may act as both tumor suppressor and oncogene, and this may partially reflect the dual roles of cell senescence at the gene level. However, a majority of the oncogenes (39/42) overlapping with cell senescence are not related to tumor suppression. In summary, although cell senescence was thought to suppress tumor development, the potential oncogenic role of cell senescence deserves further systematic evaluation.

Among the 92 cancer studies, we found 7 studies on SCC that had a relatively high mutational rate of >62.2%. All 7 of these studies are ranked in the top 16 mutated cancer studies. Using the hypergeometric test with the 92 studies as background, the probability of drawing 7 successes or more from a sample of 16 is 1.30E−5. The SCCs are cancers of the squamous cells, a kind of epithelial cell. The SCCs have histological distinctions from other cancers, such as the presence of keratin, tonofilament bundles, or desmosomes. The lack of structural protein is associated with cell-to-cell adhesion. SCC can occur in diverse tissues, including the cervix, esophagus, lips, lung, mouth, prostate, skin, urinary bladder, and vagina. SCC of the skin is the most common among all sites of the body. As one of the major forms of skin cancer, it was estimated that there were 180 000 to 400 000 cases of cutaneous SCC (skin cancer) in the United States in 2013.^33^ More interestingly, cutaneous SCC occurs more often in older people, with a peak incidence at ∼60 years. Our results may provide a clue for further exploration of the role of cell aging in SCC cancers.

In the future, we will continue to maintain and update the CSGene database once new data become available, especially data for long noncoding RNA, proteomics, epigenomics, and metabolomics data. We also plan to collect more useful annotations and add homologous genes for other model species. Our aim is to present a continuously updated, high-quality, and reference-based database to facilitate cell senescence studies.

## Materials and Methods

### Biological functional annotations and database construction

We retrieved comprehensive functional information from public resources to present the biological functions involved and overrepresented in our 504 cell senescence genes. The basic gene information and sequences were included and crosslinked to the NCBI Entrez gene,^34^ UniProt,^35^ Ensembl,^36^ and Gene Ontology databases.^37^ The mRNA expression profiling data from both normal and tumor tissues were imported from BioGPS.^38^ To obtain comprehensive pathway-related information, we annotated the cell senescence genes using the transporter substrate database,^39^ BioCyc,^40^ KEGG Pathway,^41^ rate-limiting enzyme database,^42^ PANTHER,^43^ PID Curated,^44^ the pathway localization database,^45^ PID, and Reactome.^46, 47^ The diseases involved were incorporated from GAD (gene association database),^48^ KEGG Disease,^49^ Fundo,^50, 51^ NHGIR,^52^ and OMIM.^34^ In addition, the PubMed literature associated with cell senescence was hyperlinked to each gene. Using a Perl Script and Swiss knife module,^53^ a semiautomatic annotation pipeline was implemented to integrate functional information from Gene annotation,^54^ Gene Ontology annotation, HPRD/BIND/BioGRID interaction annotation,^55, 56, 57^ KEGG LIGAND/BioCarta signaling event annotation,^58, 59^ and OMIM annotation.^60^

To obtain updates of relevant literature in the future, we constructed an automatic literature searching term using the ‘My NCBI' tool that will return matching literature every 2 weeks. We will consider using the Entrez literature similarity to cluster the newly available literature to help with literature curation. In addition, to keep pace with the fast generation of cancer genomic data, we have implemented an automatic system to import functional information from a variety of public data sources that can help to integrate more annotations quickly. Once the data content is updated, the web interface will accordingly be updated annually.

### Gene set enrichment analysis

The functional enrichment analysis in this study for pathway, disease, and other functional annotations for each gene set were conducted using ToppFun.^61^ In these enrichment analyses, all of the human protein-coding genes were used as a background to calculate statistical significance using a hypergeometric model. The Benjamini–Hochberg multiple testing corrected *P*-values for enriched annotations were also used based on a hypergeometric test using ToppFun. Finally, the enriched annotations with corrected *P*-values <0.01 were identified as overrepresentative annotations for each gene set. The resulting Gene Ontology enrichment results were further visualized by the REVIGO online server.^62^

### Gene ranking using ToppGene and cancer mutation landscape

Using the ToppGene gene ranking tool,^61^ we prioritized the gene importance for all 504 genes in CSGene. ToppGene uses integrated biological annotation data to rank the input genes, including protein domain, Gene Ontology evidence, pathway annotations, gene coexpression, sequence features, and literature mining data. However, ToppGene requires a training gene set that includes reliable genes to the biological process of interest. The training set was defined as 14 genes (*TP53*, *CDKN2A*, *CDKN1A*, *TMEM140*, *SIRT1*, *TERT*, *LMNA*, *MAPK14*, *MYC*, *MTOR*, *RB1*, *PML*, *BRAF*, and *TGFB1*) with more than five pieces of evidence from the literature. This training set was used to extract features shared by all of the cell senescence genes. Next, ToppGene builds a ranking model based on the extracted biological features from the training set. The ToppGene ranking model was then used to prioritize the remaining 489 genes using multiple dimensional data. Finally, the ToppGene ranking model combines all the rankings into a global ranking for all of the candidate cell senescence genes using order statistics (Supplementary Table S5). The top 100 ranked cell senescence-related genes were input into the cBio portal to obtain a mutation pattern across multiple cancers.^63^ A total of 716 human tumor suppressor genes were downloaded from the TSGene database,^14^ and 295 human oncogenes were collected from our previous study.^64^

### Constructing a protein–protein interactome for cell senescence genes

To connect all cell senescence genes in a pathway context, we extracted PPIs between 504 cell senescence genes and other human genes. This provides a broader biological picture associated with cell aging. To this end, a non-redundant pathway-based human interactome was built based on all of the known PPIs in PathCommons. The PathCommons database collects PPIs from pathway databases, including HumanCyc, the NCI signaling pathway database, Reactome, and KEGG. The final human pathway-based interactome contains 3629 genes and 36 034 interacting edges. To extract a subnetwork associated with the 504 cell senescence genes, we used a similar approach to one implemented in our previous study.^31^ This algorithm mapped all interesting input genes to the human interactome, and then it generated a subnetwork with the shortest paths between input genes and other genes.

Because of the complexity of a biological network, it is difficult to study the gene functions one by one. However, a network often follows a few simple rules that may relate to its function.^65^ The topological properties of networks are generally used to reveal their potential function. Therefore, topological analyses were conducted using the NetworkAnalyzer plugin in Cytoscape 2.8^66^ (Figures 6b and c) to decompose the reconstructed interactome of cell senescence genes. The degree is often used to indicate the amount of connections for each node in a network.^65^ The short path is used to characterize the shortest steps for one node to reach another.^65^ The final network visualization was performed using Cytoscape 2.8.^66^

### Web interface development

**Figure 7:**
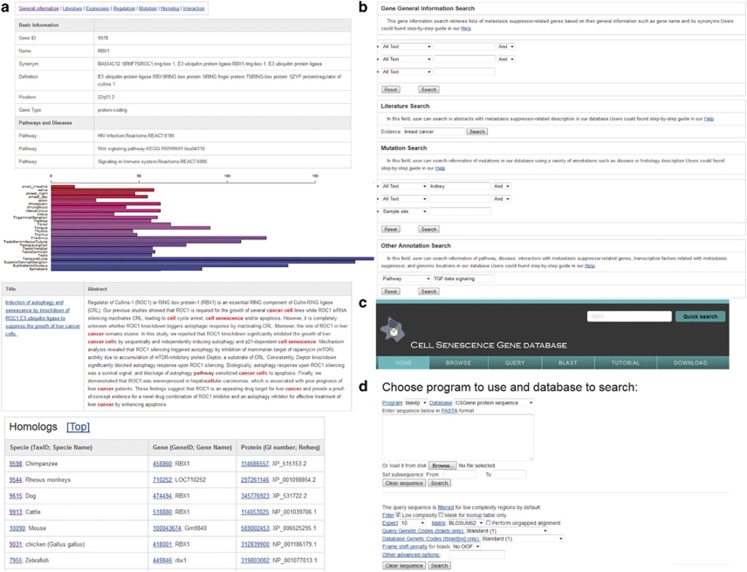
Web interface of CSGene database. (**a**) Basic information on each cell senescence gene page. (**b**) Query interface for text search. (**c**) BLAST search interface for comparing query against all sequences in CSGene. (**d**) Gene browsing interface in CSGene

To provide public access to CSGene, we used MySQL as a reliable relational database management system to store all the data and annotations on a Linux server. The Perl-based Common Gateway Interface (CGI) was used to generate dynamic contents on web pages. JavaScript and Cascading Style Sheet (CSS) technologies were used to lay out the contents in web pages for each gene. All genes in CSGene are hyperlinked to other biological annotation data resources such as gene resource for epithelial mesenchymal transition^67^ and metastasis suppressor database^68^ (Figure 7a). The comprehensive gene expression profiles related to normal tissues and cancer samples from BioGPS^38^ are represented in colored bar plots (Figure 7a). The PubMed abstracts associated with cell senescence genes are colored with keywords related to cell senescence or diseases.

The user-friendly web interface was implemented to perform text queries (Figure 7b) or to run a sequence similarity search (BLAST) against all the nucleotide and protein sequences in CSGene (Figure 7c). On the text-based query page, four useful forms are provided for the Entrez Gene ID, pathway, and disease information, as well as the genomic location, literature evidence, and mutations in cancer samples. To provide a convenient search, we presented a quick text search box for GeneID and a gene symbol at the top right of each page. Users can explore the data in CSGene in various ways, including significantly enriched pathways, related diseases, and chromosome numbers (Figure 7d). For each involved KEGG pathway, a colored pathway map is drawn to highlight the location of related cell senescence genes on the map. For large-scale bioinformatics analysis, we provide a downloadable file containing gene sequence information in plain text for all 504 integrated cell senescence genes.

## Figures and Tables

**Table 1:** The statistically significant enriched pathways of cell senescence genes

**Pathway**	**Adjusted** ***P*****-values**^a^
RNA Polymerase I Promoter Opening	3.79E−79
Meiosis	4.45E−74
Meiotic Recombination	5.38E−74
Packaging of Telomere Ends	6.00E−67
Cell Cycle	3.64E−65
Amyloids	6.25E−64
RNA Polymerase I Transcription	1.15E−62
RNA Polymerase I Chain Elongation	1.59E−62
RNA Polymerase I Promoter Clearance	5.88E−62
Meiotic Synapsis	2.35E−54
Telomere Maintenance	1.21E−51
RNA Polymerase I, RNA Polymerase III, and Mitochondrial Transcription	5.76E−50
Deposition of New CENPA-containing Nucleosomes at the Centromere	6.24E−49
Nucleosome assembly	6.24E−49
Chromosome Maintenance	1.26E−48
Alcoholism	3.78E−44
Progesterone-mediated oocyte maturation	2.01E−29
TGF-*β* Receptor Signaling Pathway	2.87E−28
APC/C:Cdc20 mediated degradation of Cyclin B	2.72E−27
Regulation of Telomerase	8.83E−27

a Adjusted *P*-values: the *P*-values of the hypergeometric test were corrected by Benjamini–Hochberg multiple testing correction.

**Table 2:** The significantly enriched diseases of cell senescence genes

**Pathway**	**Adjusted** ***P*****-values**^a^
Transcriptional activation of the integrated chromatin-associated human immunodeficiency virus type 1 promoter	2.54E−80
Regulation of HIV-1 gene expression by histone acetylation and factor recruitment at the LTR promoter	2.74E−78
Enhancement of the p300 HAT activity by HIV-1 Tat on chromatin DNA	3.21E−76
Acetylation of HIV-1 Tat by CBP/P300 increases transcription of integrated HIV-1 genome and enhances binding to core histones	5.17E−68
Systemic lupus erythematosus	5.84E−43
Viral carcinogenesis	9.94E−40
Integrated Pancreatic Cancer Pathway	5.57E−34
Pathways in cancer	2.37E−29
Hepatitis B	2.11E−28
Signaling Pathways in Glioblastoma	2.44E−28
HTLV-I infection	1.24E−27
Pancreatic cancer	8.83E−27
Breast Neoplasms	2.17E−25
Prostate Cancer	2.00E−21
Chronic myeloid leukemia	3.42E−21
Carcinoma, Hepatocellular	3.47E−19
Bladder cancer	1.25E−18
Integrated Breast Cancer Pathway	1.38E−18
MicroRNAs in cancer	1.61E−18
Colorectal cancer	1.28E−17

a Adjusted *P*-values: the *P*-values of the hypergeometric test were corrected by Benjamini–Hochberg multiple testing correction.

**Table 3:** The top 20 ranked cell senescence genes

**Gene symbol (ranked)**	**Global rank** ***P*****-value**^a^	**Global rank ratio**^b^
*CDKN2B*	2.40E−10	0.683162791
*CCND1*	5.10E−10	0.759504452
*HDAC1*	1.08E−09	0.658433129
*E2F1*	1.09E−09	0.690059176
*E2F3*	1.76E−09	0.710810668
*CCNE1*	2.38E−09	0.72747951
*JUN*	2.68E−09	0.677420458
*PCNA*	3.56E−09	0.810169252
*SMAD3*	3.97E−09	0.655630099
*MAPK1*	1.06E−08	0.720590265
*SIRT6*	1.09E−08	0.63268531
*TGFB2*	1.13E−08	0.723337593
*SMAD1*	1.37E−-08	0.651638075
*CDKN2C*	1.67E−08	0.653713064
*AKT1*	1.68E−08	0.737075122
*CCNE2*	1.73E−08	0.692954436
*TP63*	1.87E−08	0.736742708
*PRKDC*	2.42E−08	0.717291837
*RBL1*	3.11E−08	0.729568657
*MAPK3*	3.34E−08	0.691720353

a Represents the probability that a candidate gene would obtain these ranks by chance.

b The overall ranking ratio from ToppGene.

## Abbreviations

CSGene: cell senescence gene
TSGene: tumor suppressor gene
TCGA: The Cancer Genome Atlas
